# Implementation of changes in perinatal care in the North of the Netherlands, the ACTion project

**DOI:** 10.1186/1472-6963-14-S2-P31

**Published:** 2014-07-07

**Authors:** Andrea Drost, Gera Welker, Jan-Jaap Erwich

**Affiliations:** 1 Department of Obstetrics. University Medical Centre Groningen (UMCG), Groningen, The Netherlands; 2 Knowledge Center Quality & Safety UMCG, Groningen, The Netherlands

## Background

Perinatal audits are used to discuss the causes of stillbirth and neonatal deaths. These audit meetings are organized in every hospital and attended by mid wives, gynecologists, nurses, pediatricians, and managers from hospital and maternity care organizations at least twice a year.

Every audit group formulates its own local improvements. Unfortunately it appears that not all improvements are implemented properly. Therefore, to foster implementation skills of professionals that are involved, the project ‘Audit generated changes in perinatal care using tailored implementation strategies’ (ACTion) started in 2013. The objective of the project is to improve implementation skills of professionals working in perinatal care to enhance changes that were identified during audits.

## Materials and methods

The project covers the northern region of the Netherlands, in which there are 11 groups who execute perinatal audits. We offer implementation training on the spot to these professionals in 3 consecutive time periods. During the training we introduce the implementation methodology according to Grol et al [1], as well as the basics of change knowledge, which is subsequently applied on local improvement projects. All groups are offered 3 guided follow-up meetings per year to support further implementation and changing skills. The training is evaluated through standardized evaluation forms. The progress of the groups as well as the effectiveness of the program is monitored by questionnaires, observation, process journals, and interviews.

## Results

Since the start of the project, 11 groups have been trained. The size of the groups varies between 4 and 10 people, resulting in a total of 78 professionals with 7 different backgrounds. The groups have applied the newly learned implementation method to 22 improvement items in the first year of the project.

The pre and post self-assessment of the training showed an increase in implementation knowledge. The training also resulted in changes in the audit meetings regarding formulating improvements and assigning responsibility for implementation to specific professionals. Feedback on the implementation progress of improvements is increasingly included in the auditing process.

## Conclusions

After the 3-sessions training, all involved professionals were more knowledgeable on the implementation method and all were positive on their increased ability to incorporate improvements derived from perinatal audits. Moreover this ability enhances restructuring the discussion and feedback on improvement projects during the audit meetings.

## References

[B1] Grol R Wensing M Eccles M Davis D ‘Improving Patient Care. The Implementation of Change in Healthcare’ 2013 John Wiley & Sons

